# Reduction of *Salmonella* Typhimurium by Fermentation Metabolites of Diamond V Original XPC in an *In Vitro* Anaerobic Mixed Chicken Cecal Culture

**DOI:** 10.3389/fvets.2016.00083

**Published:** 2016-09-16

**Authors:** Peter Rubinelli, Stephanie Roto, Sun Ae Kim, Si Hong Park, Hilary O. Pavlidis, Don McIntyre, Steven C. Ricke

**Affiliations:** ^1^Department of Food Science, Center for Food Safety, University of Arkansas, Fayetteville, AR, USA; ^2^Diamond V, Cedar Rapids, IA, USA

**Keywords:** *Salmonella* Typhimurium, Diamond V Original XPC, mixed anaerobic culture, *in vitro*, reduction, short chain fatty acids

## Abstract

Fermentation metabolites of Diamond V Original XPC™ (XPC), a biological product derived from yeast fermentation, were evaluated for their ability to reduce the *Salmonella* Typhimurium population using an *in vitro* mixed anaerobic culture system containing cecal microbiota to simulate chicken hindgut conditions. Four different samples were prepared: anaerobic mixed culture containing (1) feed only, (2) cecal only (ceca were harvested from 42 days old broiler chickens), (3) feed and cecal contents, and (4) feed, cecal contents, and 1% XPC. Two experimental conditions were investigated: Group 1, in which the cecal content was added at the same time as a *S*. Typhimurium marker strain and Group 2, in which the cecal content was preincubated for 24 h prior to the inoculation with the *S*. Typhimurium marker strain. The mixed cultures were incubated anaerobically at 37°C, and the *S*. Typhimurium marker strain was enumerated at 0, 24, and 48 h. Analysis of short chain fatty acids was also conducted for 24 h. In the Group 1 experiment, adding XPC did not exhibit significant reduction of *S*. Typhimurium. However, the presence of XPC resulted in rapid reduction of *S*. Typhimurium in Group 2. *S*. Typhimurium was reduced from 6.81 log_10_ CFU/ml (0 h) to 3.73 log_10_ CFU/ml and 1.19 log_10_ CFU/ml after 24 and 48 h, respectively. These levels were also 2.47 log_10_ and 2.72 log_10_ lower than the *S*. Typhimurium level recovered from the control culture with feed and cecal contents, but without XPC. Based on these results, it appears that the ability of XPC to reduce *S*. Typhimurium requires the presence of the cecal microbiota. Short chain fatty acid analysis indicated that acetate and butyrate concentrations of cultures containing XPC were twofold greater than the control cultures by 24 h of anaerobic growth. Results from the present study suggest that dietary inclusion of XPC may influence cecal microbiota fermentation and has the potential to reduce *Salmonella* in the cecum. Implications of these findings suggest that XPC may decrease preharvest levels of *Salmonella* in broilers and layers.

## Introduction

Food-borne disease continues to be one of the primary public health concerns throughout the world. Infections by *Salmonella* are one of the leading causes of food-borne gastroenteritis to systemic infections in humans. Annually, it is estimated that over one million Americans contract *Salmonella* (1), and yearly costs for *Salmonella* control efforts are estimated to be up to $14.6 billion (2, 3). Salmonellosis usually occurs by consumption of foods or water contaminated with *Salmonella*, and common sources are poultry and poultry products (4), thus it is essential to control pathogenic *Salmonella* in poultry products.

Because the use of antibiotic growth promoters provoke a negative reaction from many consumers due to public health concerns such as the appearance of antibiotic resistance, the food industry has been searching for effective alternatives to replace antibiotics (5–7). Prebiotics can be defined as non-digestible food ingredients that selectively simulate the growth of beneficial bacteria and/or minimize pathogen growth in the colon, and they are occasionally used in the poultry industries to improve poultry health as a replacement of antibiotic growth promoters (8–11).

However, there are several ingredients that do not stringently fit the definition of prebiotics, but nevertheless provide similar and beneficial effects on host health with different modes of action compared to prebiotics. These ingredients are referred as “prebiotic-like compounds” (12). Fermentation metabolites of Diamond V Original XPC™ (XPC; Diamond V, Cedar Rapids, IA, USA) is a common prebiotic-like compound, which includes post-fermentation growth medium residues, residual yeast cells, and yeast cell wall fragments (mannan-oligosaccharides and β-glucans) (13). To date, several studies of XPC have focused on its effects on the host including feed uptake, growth performance, reproductive performance, and immunomodulatory functions with different animal model systems (13–17); however, few studies have examined inhibitory/bactericidal effects against pathogenic *Salmonella* (18, 19).

Because the environment of the chicken gut is anaerobic, the *in vitro* methodology using an anaerobic mixed culture can provide more empirical data since it can mimic the chicken cecal environment effectively while minimizing confounding host variables and is considered cost-effective (20). The gut microbiota ferment non-digestible ingredients to produce various compounds including short chain fatty acids (SCFA), methane, hydrogen, and ammonia (21). Among these, SCFA are potential metabolites that can be inhibitory to pathogens such as *Salmonella* (22, 23). In the present study, the ability of XPC in feed to reduce *S*. Typhimurium was investigated using a mixed anaerobic culture system to mimic conditions within the chicken hindgut. Additionally, the requirement for cecal microbiota on the reduction of *S*. Typhimurium by XPC was established. Finally, SCFA analysis was performed on the anaerobic cultures with or without XPC to further characterize the effect of XPC on cecal fermentation.

## Materials and Methods

### Preparation of Anaerobic Dilution Solution

Our *in vitro* anaerobic mixed culture experiment was based on the method of Donalson et al. The mixed cultures were grown in anaerobic dilution solution (ADS), consisting of 0.45 g/l K_2_HPO_4_, 0.45 g/l KH_2_PO_4_, 0.45 g/l (NH_4_)_2_SO_4_, 0.9 g/l NaCl, 0.1875 g/l MgSO_4_-7H_2_O, 0.12 g/l CaCl_2_-2H_2_O, 1 ml/l 0.1% resazurin, 0.05% cysteine-HCl, and 0.4% CO_2_-saturated sodium carbonate, with the sodium carbonate added last as described previously (24–29). ADS was sparged with an anaerobic gas mixture (90% nitrogen/5% carbon dioxide/5% hydrogen) for 30 min in an anaerobic chamber using an aquarium air pump and airstone prior to autoclaving. Autoclaved ADS was cooled to room temperature and allowed to equilibrate overnight in an anaerobic chamber (Coy Laboratories, Grass Lake, MI, USA) with the same atmosphere described above to remove all traces of oxygen.

### Bacterial Culture

*Salmonella* Typhimurium marker strain ST97, a nalidixic acid-resistant (NA^R^) isolate (gift of Dr. Billy Hargis, Poultry Health Laboratory, University of Arkansas) was used in the present study. This isolate was grown in sterile glass culture tubes with agitation for 16 h in Luria–Bertani (LB) medium containing 20 μg/ml nalidixic acid, 37°C at 250 rpm. The bacterial suspension was washed three times in phosphate-buffered saline (PBS).

### Cecal Sample Preparation

Ceca from three different CO_2_-euthanized 42-day-old Cobb male broiler chickens (Cobb-Vantress, Siloam Springs, AR, USA) were collected separately using alcohol-dipped, flame-sterilized tools. A University of Arkansas Institutional Animal Care and Use Committee (IACUC)-approved protocol was used to ensure humane treatment of the chickens (IACUC # 15052). Ceca were placed in sterile sample bags (VWR, Radnor, PA, USA). The bags were then placed in a portable anaerobic box (Mitsubishi Gas Chemical Co., Japan) containing oxygen-scrubbing sachets. Immediately after harvest, ceca were transferred to an anaerobic chamber (Coy Laboratory Products, Grass Lake, MI, USA). Two palladium catalyst scrubbers running continuously maintained an anaerobic environment inside the chamber.

### Anaerobic *In Vitro* Mixed Cultures

A portion of the cecal contents from three individual chickens were each removed aseptically within the chamber, weighed, and diluted 1:3000 by addition of 0.1 g of cecal content to 300 ml ADS for each chicken. A total of 20 ml of this diluted cecal content was transferred to each serum bottle with or without ground chicken feed (40 mesh) and XPC as indicated below. An additional culture received sterile ADS, but no cecal content. An initial inoculum of approximately 1 × 10^7^ CFU/ml of *S*. Typhimurium was added to each 20 ml culture. Cultures were stoppered with airtight rubber stoppers and aluminum crimps, removed from the anaerobic chamber, and incubated at 37°C with 150 rpm shaking for 48 h.

**Figure 1:**
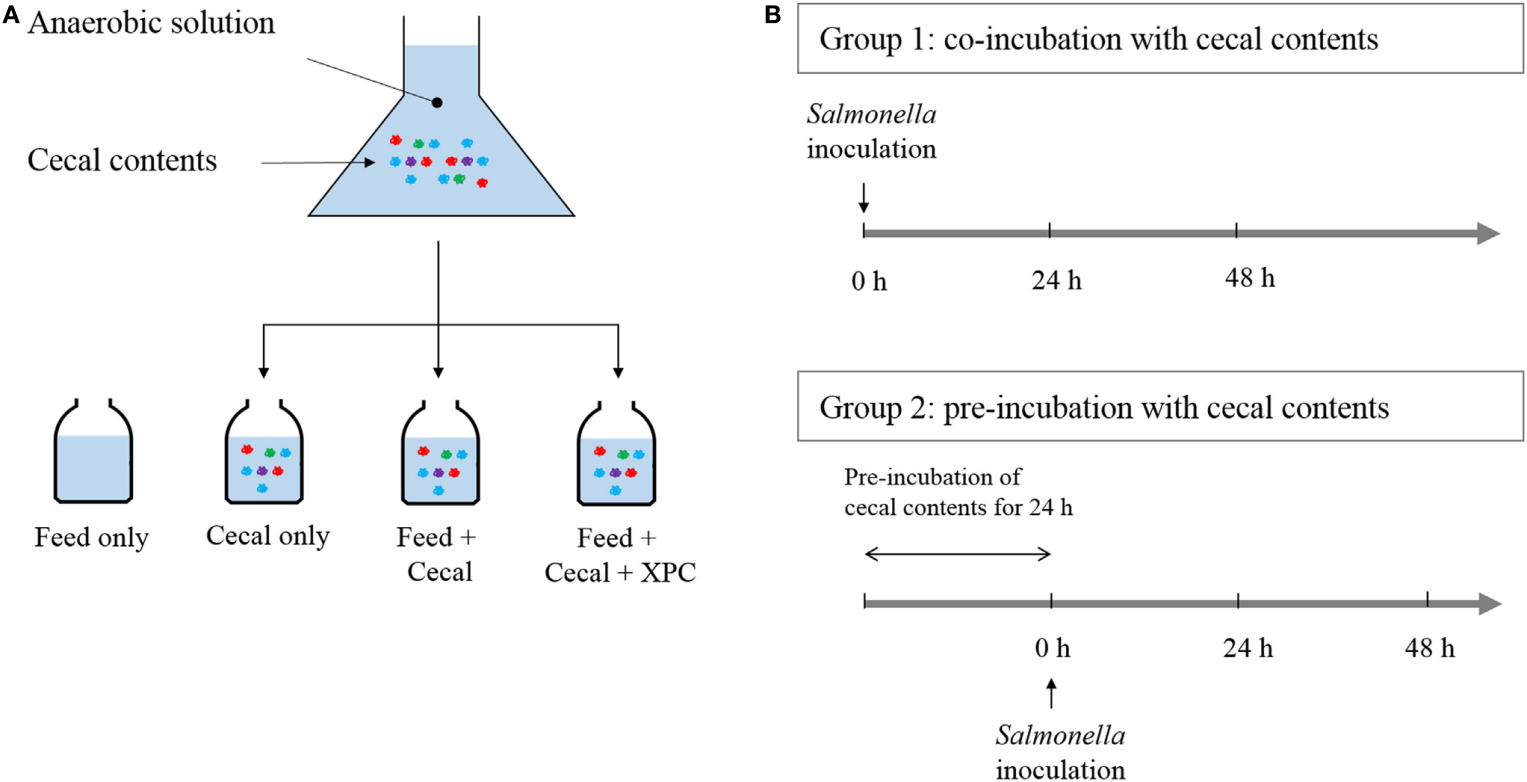
**Anaerobic experimental strategy**. **(A)** Cecal content is added to anaerobic solution with chicken feed and fermentation metabolites of Original XPC. Controls contain (1) chicken feed, but no cecal content; (2) cecal content, but no chicken feed and; (3) feed + cecal content, respectively. Experimental treatments contain feed, cecal contents, and XPC. **(B)** Group 1 cultures receive *Salmonella* at the same time as cecal content (at 0 time). Group 2 cultures receive *Salmonella* after a 24 h incubation of cecal content under anaerobic conditions. Each culture is then incubated with *Salmonella* for 48 h.

Two different experimental designs were employed, referred to as Group 1 (unadapted) and Group 2 (adapted), respectively. The experimental designs are illustrated in Figure 1. In Group 1, the *Salmonella* NA^R^ marker strain was added at the beginning of the culture incubation along with cecal bacteria, and/or chicken feed, and/or XPC. In Group 2, *S*. Typhimurium was added after a 24 h preincubation of the cecal bacteria with the chicken feed and/or XPC. Three control cultures were run in parallel as indicated in Figure 1.

### *Salmonella* Enumeration

At 0, 24, and 48 h, an aliquot of each culture was removed, diluted, and spread on Brilliant Green Agar medium (BG, BD Biosciences, Franklin Lakes, NJ, USA) supplemented with 20 ug/ml nalidixic acid for quantitation of colony forming units (CFU) of marker strain *S*. Typhimurium per milliter of culture. The diluted cecal contents were also tested for NA^R^ bacteria prior to addition of marker strain *S*. Typhimurium by inoculation into tetrathionate (TT) enrichment broth (BD Biosciences, Franklin Lakes, NJ, USA), and none were detected. If no *Salmonella* were detected at a particular time point in undiluted culture, that culture was inoculated into TT enrichment broth to confirm that no *S*. Typhimurium survived.

### Short Chain Fatty Acid Analysis

Anaerobic culture supernatants were stored at −20°C until they could be analyzed by gas chromatography. A 1 ml portion of culture supernatant was centrifuged at 14,000 × *g* to remove solids. An aliquot of the supernatant (450 μl) was then mixed with 50 μl of GC reagent (50 mM 4-methyl-valeric acid, 5% meta-phosphoric acid, 1.6 mg/ml copper sulfate). This mixture was allowed to incubate at 25°C for 10 min and subsequently centrifuged at 14,000 × *g*. The supernatant was transferred to a fresh tube and 1 μl was loaded into a Shimadzu 2010 gas chromatograph (Kyoto, Japan) fitted with a 30 m × 0.25 mm BP21 glass capillary column with 0.25 mm film thickness (SGE, Austin, TX, USA) operated at 100 kPa He carrier gas pressure, with 170 kPa H_2_, Ar, and air pressure, at 100°C for 3 min, followed by a temperature gradient of 4°C/min to 120°C, holding at 120°C for 1 min, followed by a further gradient of 3°C/min to 150°C. The SPL was maintained at 220°C with split ratio = 30. FID was maintained at 230°C. Carrier gas flow rate was set to 30 ml/min. A 1:100 mixture of acetic, propionic, and butyric acids was serially diluted, mixed with GC reagent, and used as standards. Peak areas were normalized for loading differences using the valeric acid internal control from the GC reagent.

### Statistical Analysis

Means were determined to be significantly different if *P* < 0.05 by two-tailed paired Student *t*-test using Microsoft Excel.

## Results and Discussion

The main objective of this study was to investigate the inhibitory effect of XPC on *S*. Typhimurium when combined *in vitro* with cecal microbiota. The ceca are the main site where pathogens including *Salmonella* colonize (30). Since poultry have a relatively slow digestion transit time, poultry ceca have a large number of bacteria, and the majority of these are strictly anaerobic (27, 31, 32). Cecal bacteria in poultry become more diverse as the host matures, and they can maximize metabolic fermentation in an anaerobic environment (12). Cecal contents used in this study were obtained from mature chickens (42-day-old chickens), thus it should serve as a source of a fairly diverse microbiota containing a wide range of anaerobic bacteria. Also, using an anaerobic mixed culture in this study could help to understand the actual fate of *Salmonella* in ceca by various feeding conditions.

**Figure 2:**
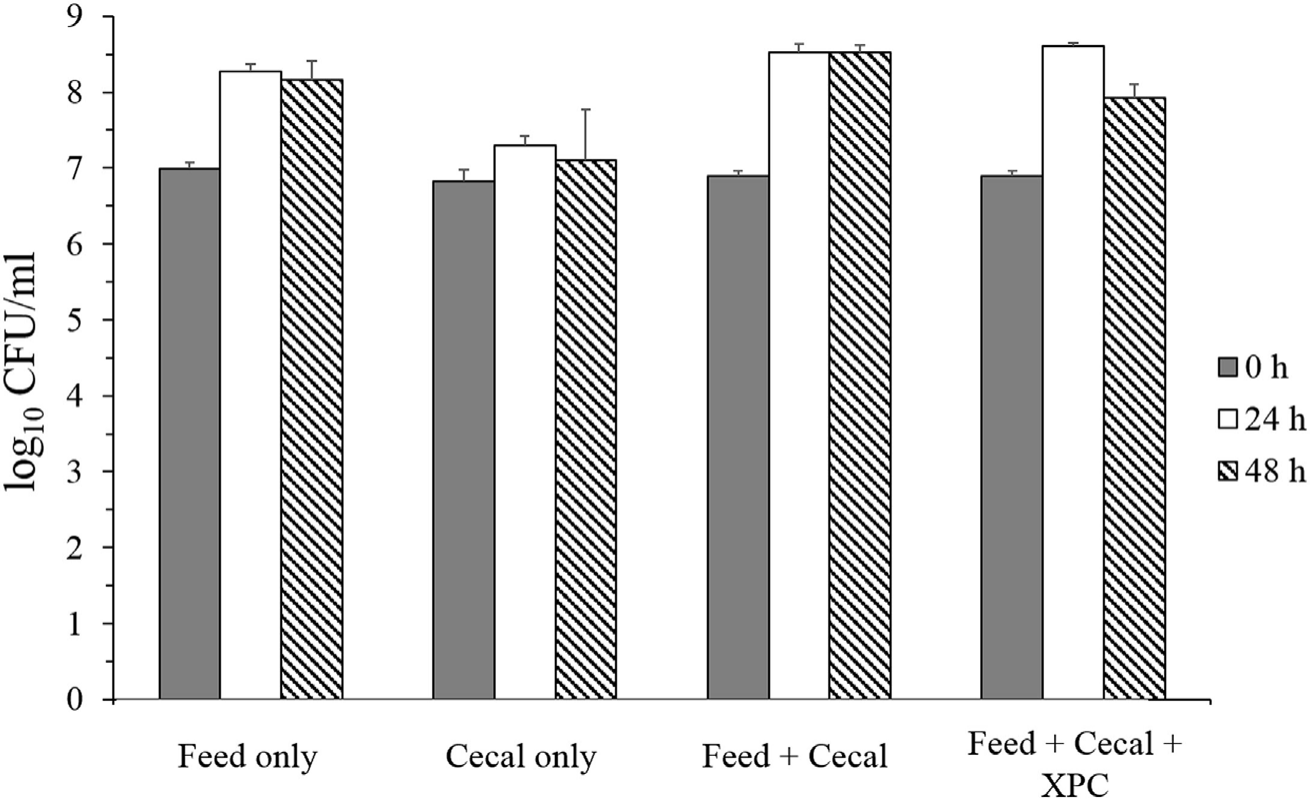
***Salmonella* Typhimurium survival in unadapted anaerobic cultures (Group 1) with and without fermentation metabolites of Original XPC**. Bars and brackets represent the mean and SE of three biological replicates.

Two conditions were investigated in the present study: Group 1 (unadapted), in which the cecal microbiota was added at the same time as the *S*. Typhimurium and Group 2 (adapted), in which the cecal microbiota was allowed to metabolize anaerobically for 24 h prior to the inoculation of *S*. Typhimurium (see Figure 1 for design). Results on *S*. Typhimurium reduction by XPC were different between groups. In the unadapted condition (Group 1), the population of *S*. Typhimurium was slightly increased or maintained during 48 h incubation in all controls (feed only, cecal only, and feed + cecal) and treatment (feed + ceca + XPC); the population after incubation was not significantly different from the initial population (Figure 2). In the feed + cecal sample, *S*. Typhimurium populations were increased from 6.89 log_10_ CFU/ml to 8.52 and 8.53 log_10_ CFU/ml after 24 and 48 h, respectively (*P* < 0.05). When XPC was added to the feed + cecal sample, *S*. Typhimurium populations were increased from 6.89 log_10_ CFU/ml to 8.60 and 7.92 log_10_ CFU/ml after 24 and 48 h (*P* < 0.05), respectively, indicating that XPC had little or no effect on *Salmonella* survival when *S*. Typhimurium was added at the same time as cecal contents.

**Figure 3:**
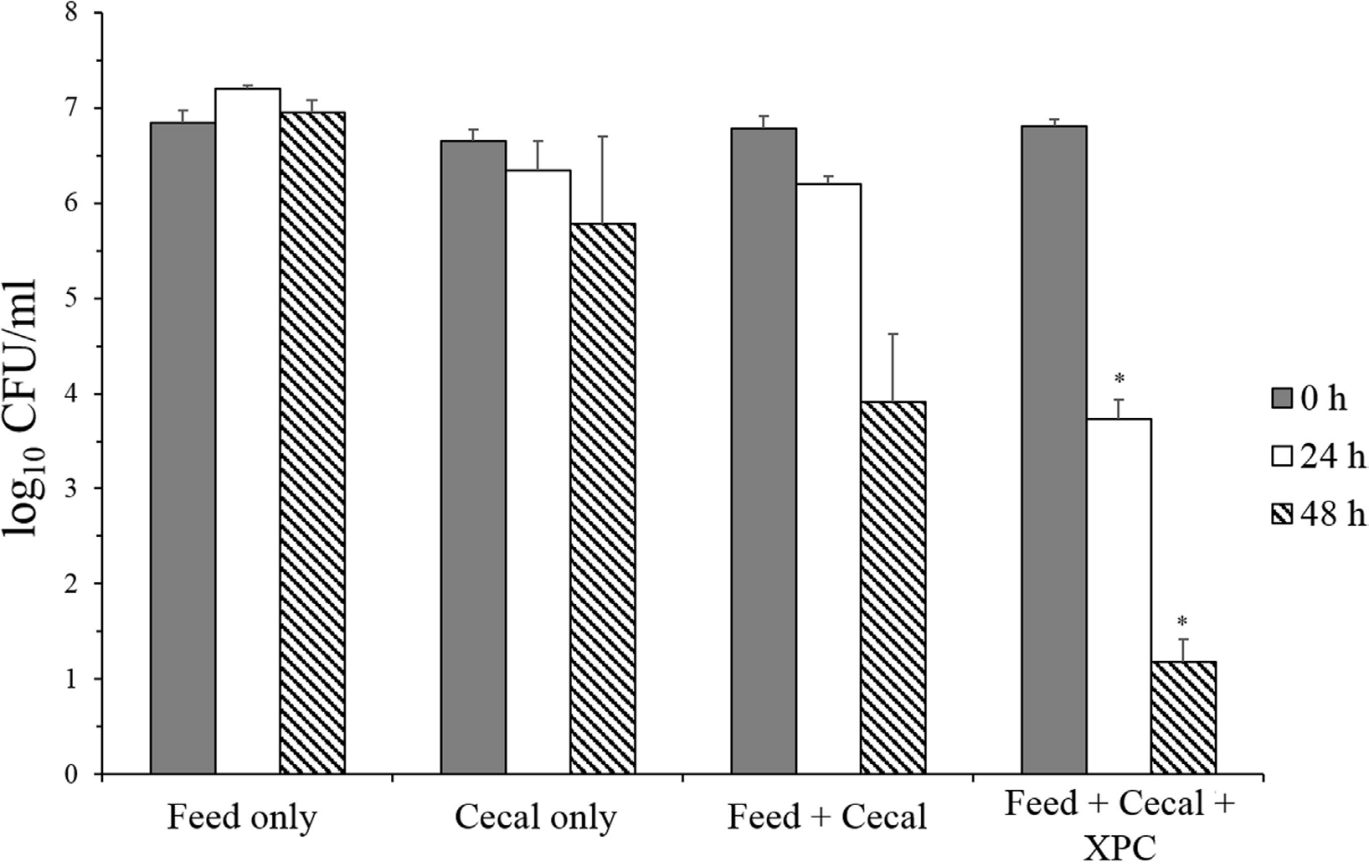
***Salmonella* Typhimurium survival in adapted anaerobic cultures (Group 2) with and without fermentation metabolites of Original XPC**. Bars and brackets represent the mean and SE of three biological replicates. Asterisks indicate significant difference *(P* < 0.05) from the “feed + cecal” control.

In contrast, XPC-containing cultures exhibited a significant reduction in *Salmonella* survival under adapted conditions (Group 2) (Figure 3). There was no reduction in *S*. Typhimurium in the feed-only control sample, and only a 0.87 log_10_ reduction of populations of *S*. Typhimurium was achieved after 48 h incubation in the cecal-only control sample. In addition, the *S*. Typhimurium population was decreased in both the feed + cecal control and the feed + cecal + XPC treatments. However, the presence of XPC resulted in a greater reduction of *S*. Typhimurium compared with the feed + cecal control. When *S*. Typhimurium was inoculated to the feed + cecal control, a 2.87 log_10_ reduction in the bacterial population was observed after 48 h. With XPC, the log_10_ reductions achieved after 24 and 48 h incubation were 3.08 and 5.62 log_10_ reduction, respectively. These levels are 2.47 log_10_ (24 h) and 2.72 log_10_ (48 h) lower than the *Salmonella* level recovered from the feed + cecal control.

**Figure 4:**
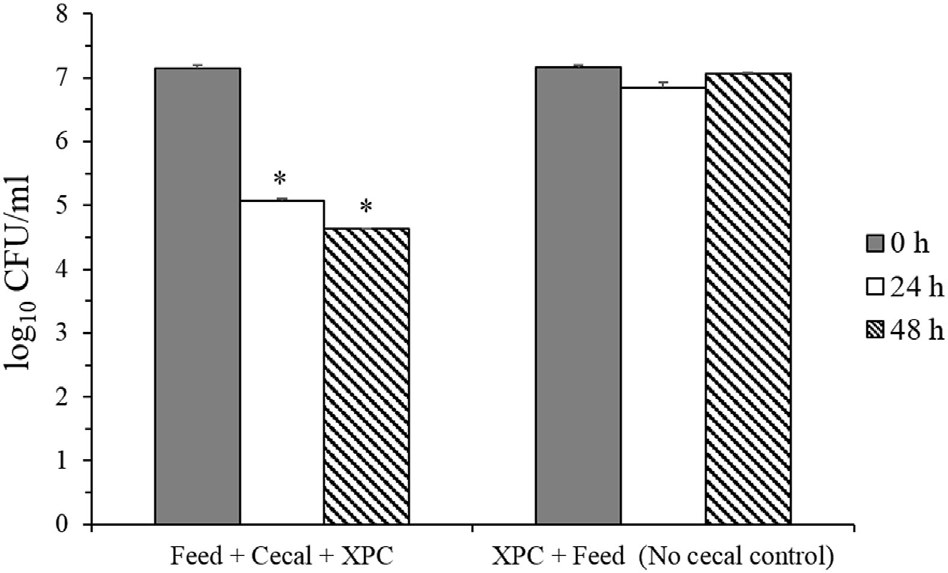
***Salmonella* Typhimurium survival in feed + cecal + fermentation metabolites of Original XPC and in control anaerobic cultures lacking cecal contents**. Asterisks indicate significant difference (*P* < 0.005) from the corresponding control.

These results suggested that adaptation of the cecal microbiome in the *in vitro* mixture to XPC prior to inoculation of *S*. Typhimurium appears to generate a more inhibitory environment for *Salmonella* than XPC unadapted cecal cultures. To evaluate the role of the microbiota on reduction of *S*. Typhimurium, survival of *S*. Typhimurium in “Feed + cecal + XPC” and “Feed + XPC without cecal contents” were compared (Figure 4). When *S*. Typhimurium was exposed to XPC in the absence of broiler cecal content, no reduction in *S*. Typhimurium was observed, suggesting that XPC acts in concert with cecal microbiota to inhibit *S*. Typhimurium (Figure 4). This is a further indication that cecal microbiota are essential to the reduction of *Salmonella* by XPC. These results are in accordance with a previous study reporting higher inhibitory activities of fructooligosaccharide in samples preincubated with cecal microbiota prior to inoculation of bacteria (25). Furthermore, the results from both *in vitro* studies suggest that dietary inclusion of XPC may influence cecal microbiota fermentation and has the potential to reduce *Salmonella* colonization in the cecum.

**Figure 5:**
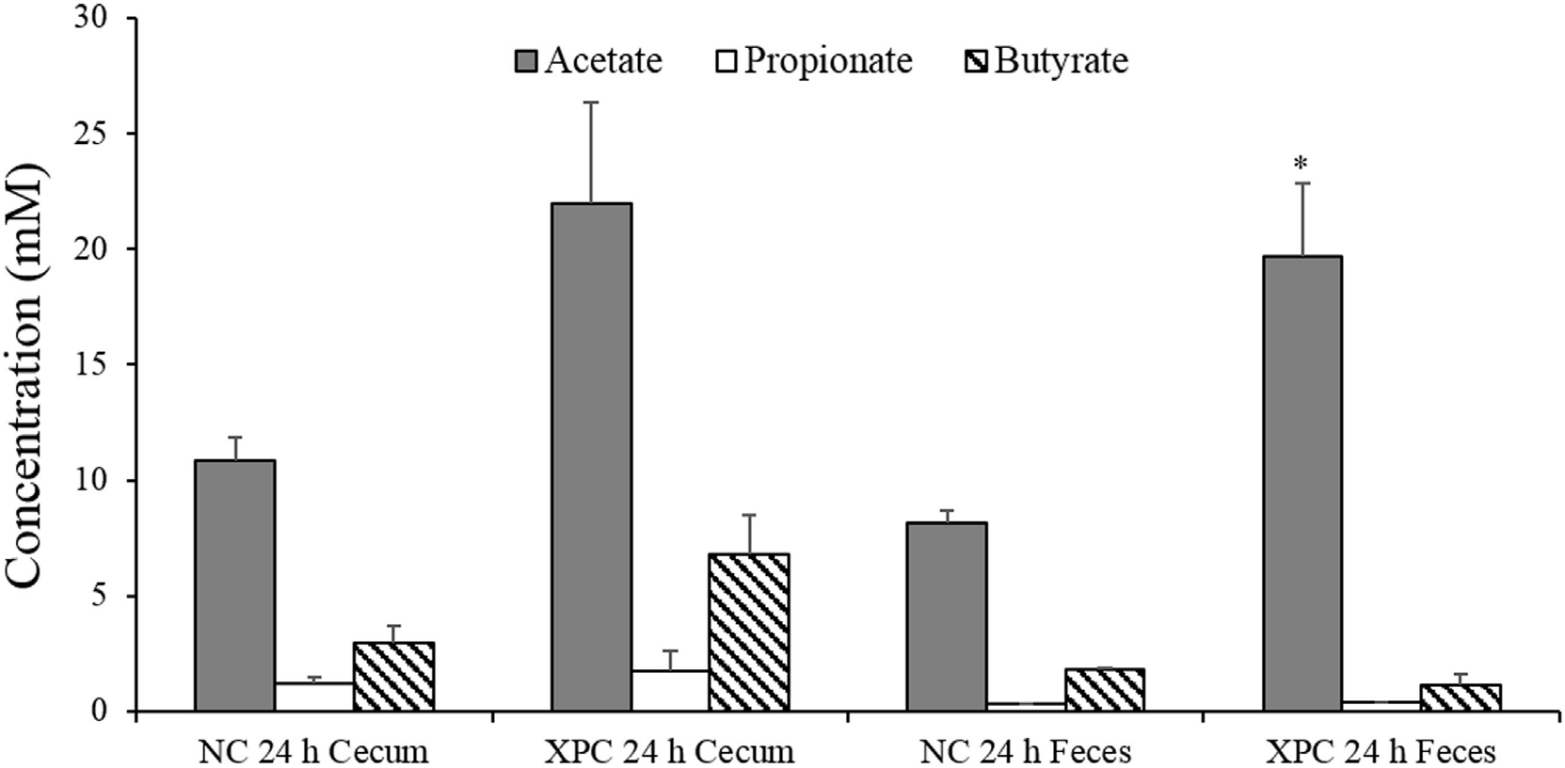
**Short chain fatty acid analysis of 24 h anaerobic cultures containing 6-week-old broiler cecal contents or feces with and without 1% fermentation metabolites of Original XPC**. Bars and brackets represent the mean and SE of three biological replicates (chickens). Asterisk indicates significant difference (*P* < 0.05) from corresponding negative control (NC).

The SCFA analysis of supernatants from the mixed cultures indicated that acetate and butyrate concentrations of cultures containing XPC + cecum and XPC + feces were twofold greater than the control cultures after 24 h of anaerobic growth (Figure 5). This suggests one or more microorganisms have potentially increased acetate and/or butyrate production as a result of being exposed to components of XPC. This additional acetate and butyrate may be contributing to the inhibition of *Salmonella* due to the direct toxic effect of intracellular anion accumulation when these acids dissociate in the cytosol of sensitive bacteria such as *Salmonella* (22, 23, 33). Interestingly, butyrate has been found to inhibit *Salmonella* invasion of host cells by downregulating *Salmonella* pathogenicity island 1 (SPI-1) gene expression (22, 34). Along these lines, Feye et al. has shown that XPC fed to broilers reduces the virulence regulatory gene *hilA* in the intestine (19).

In conclusion, XPC can effectively reduce *S*. Typhimurium survival (5.62 log_10_ reduction) in an *in vitro* anaerobic mixed cecal culture, and XPC and cecal microbiota are both required for the reduction of *S*. Typhimurium survival. Incubation of cecal microbiota with XPC increased SCFA levels (particularly acetate) in anaerobic cultures. The use of XPC as a prebiotic-like compound has a number of advantages for use in poultry: (1) there are no concerns over usage of antibiotics or growth promoters or the appearance of antibiotic-resistant bacteria, (2) the use of XPC is acceptable to the both industries and consumer since it is a naturally derived yeast product (also an environmentally friendly product), (3) its use by the poultry industry is also feasible because XPC was classified as generally recognized as safe (GRAS) by US FDA (13). To the best of our knowledge, this is the first study to examine the inhibitory effects of XPC in feed with an anaerobic mixed cecal inocula culture to mimic the chicken cecal environment. The implication of these findings is that XPC may decrease preharvest levels of *Salmonella* in the ceca of broilers and layers, thus it could be a suitable alternative to antibiotics currently used in poultry industries.

## Author Contributions

PR and SR performed experiments, drafted the manuscript, collected test data, and analyzed the data. SK, HP, and DM revised the manuscript. PR, SP, and SR designed the study and revised the manuscript.

## Conflict of Interest Statement

The authors declare that the research was conducted in the absence of any commercial or financial relationships that could be construed as a potential conflict of interest.
